# Neuroprotective Effect of Kaempferol Glycosides against Brain Injury and Neuroinflammation by Inhibiting the Activation of NF-κB and STAT3 in Transient Focal Stroke

**DOI:** 10.1371/journal.pone.0055839

**Published:** 2013-02-20

**Authors:** Lu Yu, Chu Chen, Liang-Fen Wang, Xi Kuang, Ke Liu, Hao Zhang, Jun-Rong Du

**Affiliations:** 1 Key Laboratory of Drug Targeting and Drug Delivery Systems of Ministry of Education, Department of Pharmacology, West China School of Pharmacy, Sichuan University “985 Projects – Science and Technology Innovation Platform for Novel Drug Development and Translational Neuroscience Center”, Chengdu, China; 2 Sichuan Academy of Chinese Medicine Sciences, Chengdu, China; 3 Luzhou Medical College, Luzhou, Sichuan, China; University of South Florida, United States of America

## Abstract

**Background:**

Ischemic brain injury is associated with neuroinflammatory response, which essentially involves glial activation and neutrophil infiltration. Transcription factors nuclear factor-κB (NF-κB) and signal transducer and activator of transcription 3 (STAT3) contribute to ischemic neuroinflammatory processes and secondary brain injury by releasing proinflammatory mediators. Kaempferol-3-*O*-rutinoside (KRS) and kaempferol-3-*O*- glucoside (KGS) are primary flavonoids found in *Carthamus tinctorius* L. Recent studies demonstrated that KRS protected against ischemic brain injury. However, little is known about the underlying mechanisms. Flavonoids have been reported to have antiinflammatory properties. Herein, we explored the effects of KRS and KGS in a transient focal stroke model.

**Methodology/Principal Findings:**

Rats were subjected to middle cerebral artery occlusion for 2 hours followed by 22 h reperfusion. An equimolar dose of KRS or KGS was administered i.v. at the beginning of reperfusion. The results showed that KRS or KGS significantly attenuated the neurological deficits, brain infarct volume, and neuron and axon injury, reflected by the upregulation of neuronal nuclear antigen-positive neurons and downregulation of amyloid precursor protein immunoreactivity in the ipsilateral ischemic hemisphere. Moreover, KRS and KGS inhibited the expression of OX-42, glial fibrillary acidic protein, phosphorylated STAT3 and NF-κB p65, and the nuclear content of NF-κB p65. Subsequently, these flavonoids inhibited the expression of tumor necrosis factor α, interleukin 1β, intercellular adhesion molecule 1, matrix metallopeptidase 9, inducible nitric oxide synthase, and myeloperoxidase.

**Conclusion/Significance:**

Our findings suggest that postischemic treatment with KRS or KGS prevents ischemic brain injury and neuroinflammation by inhibition of STAT3 and NF-κB activation and has the therapeutic potential for the neuroinflammation-related diseases, such as ischemic stroke.

## Introduction

Ischemic stroke can lead to extensive cerebral damage to gray matter and white matter and is a significant cause of morbidity and mortality in the world. Recanalization of occluded cerebral blood vessels with intervention or thrombolysis therapies is the most effective approach to ischemic stroke treatment [1]. However, early reperfusion of the ischemic brain is associated with some risks, such as fatal cerebral edema, hemorrhagic transformation, neurovascular injury, and neuronal death. Therefore, neuroprotective agents that target diverse pathophysiological events following cerebral ischemia have been widely investigated as potential therapeutic strategies for treating ischemic stroke [2].

Although complex mechanisms are involved in the pathogenesis of brain injury after cerebral ischemia, acute neuroinflammatory reactions that begin within hours are known to contribute to extensive brain injury. Resident glial activation and neutrophil infiltration from blood to ischemic brain parenchyma play pivotal roles in the inflammatory processes after ischemic stroke by inducing the activation of transcription factors, thereby producing proinflammatory mediators [3∼5]. Kinds of nerve cells, such as microglia, astrocytes and neurons, are implicated in the ischemic inflammatory response in the brain [6]. Additionally, the microvascular endothelium and vascular extracellular matrix in the ischemic territory participate in the recruitment of polymorphonuclear leukocytes (PMNs) from the circulation [7], [8]. PMN migration involves chemotaxis, adhesion to endothelial cells, the penetration of tight junctions, and migration through the extracellular matrix. Accumulating evidence suggests that neuroinflammatory processes after cerebral ischemia involve various pathways and molecules. Of these, aberrantly activated signal transducer and activator of transcription 3 (STAT3) and nuclear factor-κB (NF-*κ*B) can promote the transcription and expression of many genes that encode proinflammatory mediators, including cytokines, chemokines, adhesion molecules, and inflammatory enzymes [5]. These proinflammatory mediators not only damage neurons but also affect gliocytes, axons, and capillaries, thereby initiating secondary brain damage after ischemic stroke [6]. Therefore, STAT3 and NF-*κ*B have become potential targets for intense drug discovery and development efforts for neuroprotection against neuroinflammation-related diseases, such as ischemic stroke.

**Figure 1:**
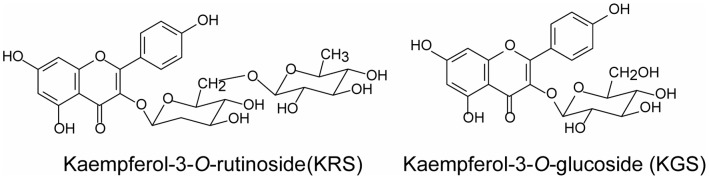
Chemical structures of kaempferol glycosides.

*Carthamus tinctorius* L. (safflower, also known as *hong hua* in Chinese) has potent activity in promoting blood circulation and removing blood stasis. It has been extensively used for the treatment of cerebrovascular and cardiovascular diseases in traditional Chinese medicine [9]. Kaempferol-3-*O*-rutinoside (KRS) and kaempferol-3-*O*- glucoside (KGS) are primary flavonoid glycosides found in *Carthamus tinctorius* L. (Figure 1). Flavonoids are naturally occurring polyphenolic compounds that contain two benzene rings linked together with a heterocyclic pyran or pyrone ring, and they are well known for their various biological activities, such as antioxidant and antiinflammatory effects [10]. Recent studies have shown that KRS improves memory dysfunction and oxidative stress in a multi-infarct dementia model and reduced ischemic brain damage by upregulating endothelial nitric oxide synthase (eNOS) activity in transient focal cerebral ischemia [11], [12]. Additionally, KGS exerted antiinflammatory effects in carrageenan-induced hindpaw edema and xylene-induced ear edema models [13]. Altogether, previous studies suggest that KRS may be a novel neuroprotectant against neurovascular injury after cerebral ischemia reperfusion by restoring cerebral blood flow and inhibiting inflammatory reactions. However, the antineuroinflammatory effect and underlying mechanism of KRS have not yet been reported.

The present study was designed to examine the neuroprotective effects of KRS and KGS on brain injury and the neuroinflammatory response after transient focal cerebral ischemia. We first prepared KRS and KGS from flowers of the safflower variety *bai hua*. To evaluate efficacy, various parameters, including neurobehavioral function, brain infarct volume, neuron and axon damage, neuropathological changes in glial cells (i.e., microglia, astrocytes, and oligodendrocytes), STAT3 and NF-κB activation, and the subsequent expression of proinflammatory mediators, were studied in a rat model of transient focal cerebral ischemia.

## Materials and Methods

### Reagents

2,3,5-triphenyltetrazolium chloride (TTC) was purchased from Sigma-Aldrich Co. (St. Louis, MO, USA). The primary antibodies used in this study, including neuronal nuclear antigen (NeuN), CD11b/c (OX-42), glial fibrillary acidic protein (GFAP), microtubule associated protein Tau-1 (Tau-1), amyloid precursor protein (APP), myeloperoxidase (MPO), inducible nitric oxide synthase (iNOS), matrix metallopeptidase 9 (MMP-9), tumor necrosis factor α (TNF-α), interleukin 1β (IL-1β), intercellular adhesion molecule 1 (ICAM-1), NF-κB p65 subunit (NF-κB p65), STAT3, phosphorylated NF-κB p65 subunit at Ser536 (p-NF-κB p65) and phosphorylated STAT3 at Tyr705 (p-STAT3), are summarized in Table 1.

**Table 1:** Primary antibodies used in this study.

Antibody	Role	Host	Dilution	Application	Source
NeuN	Neuron marker	rabbit	1∶100	IHC^a^	Bioss (Beijing, China)
OX-42	Microglia marker	mouse	1∶200	IHC	Millipore
GFAP	Astrocyte marker	rabbit	1∶100	IHC	Boster (Wuhan, China)
APP	Marker of axonal injury	rabbit	1∶100	IHC	Boster (Wuhan, China)
MPO	Neutrophil marker	rabbit	1∶100	IHC	Boster (Wuhan, China)
iNOS	Proinflammatory factor	rabbit	1∶100	IHC	Boster (Wuhan, China)
MMP-9	Proinflammatory factor	mouse	1∶200	IHC	Santa Cruz Biotechnology
TNF-α	Proinflammatory factor	rabbit	1∶100	IHC	Boster (Wuhan, China)
IL-1β	Proinflammatory factor	rabbit	1∶100	IHC	Boster (Wuhan, China)
ICAM-1	Proinflammatory factor	rabbit	1∶100	IHC	Boster (Wuhan, China)
NF-κB p65	Proinflammatory factor	rabbit	1∶1000	WB^b^	Cell Signaling Technology
STAT3	Proinflammatory factor	mouse	1∶1000	WB	Cell Signaling Technology
p-NF-κB p65	Proinflammatory factor	mouse	1∶100	IHC	Cell Signaling Technology
p-STAT3	Proinflammatory factor	rabbit	1∶200/1∶1000	IHC/WB	Cell Signaling Technology
Lamin B	Nuclear Envelope Marker	rabbit	1∶1000	WB	Boster (Wuhan, China)

a IHC, Immunohistochemistry;

b WB, Western blot.

### Animals

SPF male Sprague-Dawley rats (250–300 g body weight) were purchased from Vital River Laboratory Animal Technology Co. Ltd., Beijing, China. The animals were kept under a 12 h/12 h light/dark cycle and controlled temperature and humidity and given food and water *ad libitum*. All of the animal procedures was performed according to China Animal Welfare Legislation and approved by the Sichuan University Committee on the Care and Use of Laboratory Animals.

### Preparation of Kaempferol Glycosides

KRS and KGS were isolated from the flowers of the safflower variety *bai hua* using combined chromatography. Briefly, the flowers of safflower, cultivated at a plantation at the Sichuan Academy of Agricultural Science (Chengdu, China), were harvested in June 2010. The reference specimens were deposited at the Department of Pharmacognosy, West China School of Pharmacy, Sichuan University. The dried safflower powder was extracted with 75% ethanol (v/v) at room temperature. After solvent evaporation, the residue was dissolved in water and then subjected to a D101 macroporous adsorption resin column. Fractions were collected by increasing the ethanol content of the eluent (20–80%, v/v). Flavonoids were mainly eluted with 50% ethanol, separated on a polyamide column, and eluted with a step gradient of aqueous ethanol (10–60%, v/v). KRS and KGS were further purified from a 10% or 50% ethanol-eluted fraction by re-crystallization with ethanol or Sephadex LH-20 chromatography with 50% methanol (v/v) as the mobile phase, respectively. KRS and KGS were identified by spectroscopy, including nuclear magnetic resonance and mass spectrum analysis [14], [15]. The purities of KRS and KGS were examined based on the percentage of total peak areas by high-performance liquid chromatography (HPLC) as we described previously [16]. In the present study, KRS and KGS (purity >99%) were freshly prepared in 25% (w/w) polyethylene glycol 400 (PEG400, Tianjin Chemical Company, China).

### Induction of Focal Cerebral Ischemia-Reperfusion

Transient focal ischemia was induced by transient middle cerebral artery occlusion (tMCAO) in rats using the intraluminal filament technique [17]. The right middle cerebral artery occlusion was performed as we described previously [18]. Two hours after cerebral ischemia, the thread was carefully withdrawn to establish reperfusion. The sham-operated controls underwent similar surgical procedures without occlusion of the middle cerebral artery.

### Examination of Neurological Deficits

The neurological impairment after stroke insult was evaluated by a neurobehavioral test scored on a fivepoint scale as described previously [18]: 0, no significant deficits; 1, failure to extend left forepaw fully; 2, circling to the left; 3, failing to the left; and 4, inability to walk spontaneously combined with depressed levels of consciousness. Neurobehavioral testing was performed by one examiner blinded to the experimental groups at 2 h MCAO and 22 h reperfusion following ischemia, respectively. The rats with neurological scores <2 in the first evaluation were excluded from the present study [19].

### Drug Treatments

The tMCAO rats were randomly divided into three groups according to neurobehavioral function (*n*  = 17 each group). An equal molar dose of KRS (10.0 mg/kg) or KGS (7.5 mg/kg) was immediately administered through the tail vein injection at the beginning of reperfusion, 2 h after ischemia induction. The vehicle-treated and sham-operated groups received solvent solution in the same manner.

### Determination of Infarct Volume

After the behavioral test at 22 h after reperfusion, eight rats in each group were euthanized and the brains were rapidly removed and frozen at −20°C for 15 min. Coronal brain sections with a 2 mm thickness each were stained with a 0.5% TTC at 37°C for 15 min followed by fixation with 4% formaldehyde for 24 h. Infarct volume measurements were carried out by an investigator blinded to the treatment groups as previously described [18].

### Immunohistochemical Staining

After the behavioral test, six rats from each group were deeply anesthetized and perfused through the heart with PBS, followed by 4% paraformaldehyde. The brains were collected, and the penumbral region of coronal sections at the level of bregma approximating +0.7 to −4.3 mm in the ipsilateral ischemic hemisphere were used for the determination of immunohistochemistry as we described previously [20]. Briefly, the sections (5 µm) were rinsed with 3% H_2_O_2_ for 15 min to block endogenous peroxide activity and incubated with 5% bovine serum albumin for 30 min to block nonspecific binding. The sections then were incubated with different primary antibodies described in Table 1, respectively, overnight at 4°C. Primary antibodies were recognized by a biotinylated anti-rabbit or anti-mouse secondary antibody at 37°C for 1 h and detected by the avidin-biotin complex (ABC) kit (Boster, Wuhan, China) at 37°C for 1 h. Immunoreactions were visualized using 3,3′-diaminobenzidine tetrahydrochloride (DAB) and the nuclei were counterstained with haematoxylin. Brain sections were observed under a light microscope by an investigator who was blind to section identification. For the semiquantitative analysis of the immunohistochemical results, three sections from each brain, with each section containing three microscopic fields from the ischemic boundary zone (penumbra) in the cerebral cortex or subcortex (corpus callosum and striatum) were digitized under a 40× objective [21]. The number of neurons was calculated by counting NeuN-immunopositive cells and the other protein expressions were quantified on the basis of the integrated optical density (IOD) of the immunostained-positive cells using an image analysis system (ImagePro Plus 5.0 software, Cybernetics, Bethesda, MD). Data are presented as the number of neurons/Field or IOD of positive cells/Field.

### Western Blot Analysis

After the behavioral experiments, the ischemic cortex (*n*  = 3 each group) was collected and the total protein and nuclear protein were isolated from using RIPA buffer (Beyotime, Jiangsu, China) or a cytoplasmic and nuclear protein extraction kit (Boster, Wuhan, China) according to the manufacturer’s instructions. Equal amounts of protein were separated by 10% sodium dodecyl sulfate-polyacrylamide gel electrophoresis and transferred to a polyvinylidene fluoride membrane (Millipore, Bedford, MA, USA). The membrane was then incubated with a primary antibody described in Table 1, respectively, overnight at 4°C, followed by incubation with the appropriate horseradish peroxidase-conjugated secondary antibodies (Zhongshan-Golden Bridge, Beijing, China) for 1 h at room temperature and ECL chemiluminescence kit (Millipore, Billerica, MA, USA). The OD of each band was determined using Gel Pro Analyzer 6.0 (Media Cybernetics, Bethesda, MD, USA). The results, expressed as the ratio of p-STAT3/STAT3 and NF-κB p65/Lamin B, were normalized to protein expression in the sham-operated controls.

### Statistical Analysis

Data were expressed as mean ± SEM. Variance was analyzed by a one-way ANOVA followed by LSD test (equal variances assumed) or Dunnett's T3 test (equal variances not assumed). The neurological deficit scores were analyzed by the Mann-Whitney U test. Statistical analysis was performed with SPSS v.16.0. Values of *p*<0.05 were considered statistically significant.

## Results

### Effects of KRS and KGS on Neurological Deficit

**Figure 2:**
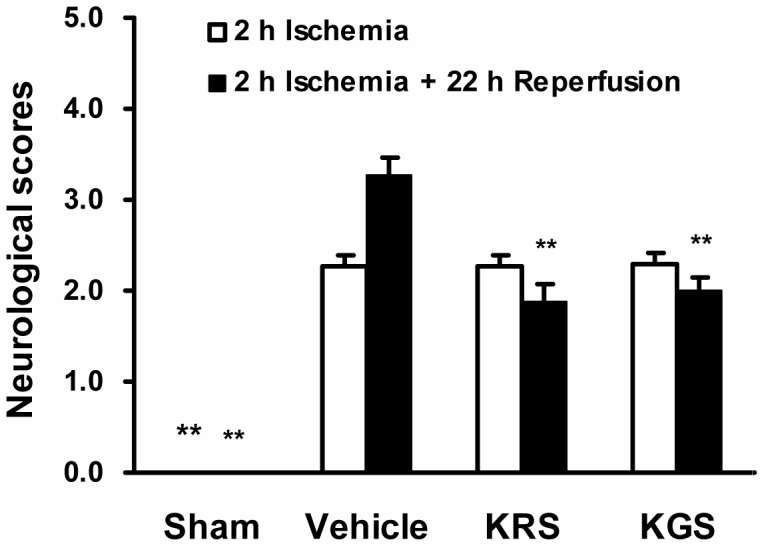
KRS and KGS improved neurological deficit in tMCAO rats. The neurological deficit scores evaluated after 2 h ischemia or 22 h reperfusion following 2 h ischemia, respectively. Post-ischemic treatment with an equimolar dose of KRS (10.0 mg/kg) or KGS (7.5 mg/kg) administrated i.v. significantly ameliorated the neurological deficit scores compared with the vehicle-treated controls. The data are expressed as mean ± SEM (*n* = 17 each group). *p<0.05, **p<0.01, compared with the vehicle-treated group.

Generally, higher neurological deficit scores were associated with more severe motor impairment. The neurobehavioral tests revealed no neurobehavioral dysfunction symptoms in the sham-operated group; therefore, these animals had a neurological score of 0. Figure 2 shows that the tMCAO rats in vehicle-, KRS-, and KGS-treated groups had similar neurological deficit scores at the end of 2 h ischemia (i.e., before treatment). At 22 h reperfusion after ischemia, however, the neurological score in the vehicle-treated group increased to 3.27±0.19, attributable to the brain injury induced by ischemia/reperfusion, whereas intravenous administration of an equimolar dose of KRS (10.0 mg/kg) or KGS (7.5 mg/kg) at the beginning of reperfusion significantly reduced the neurological scores to 1.89±0.20 and 2.00±0.14, respectively, compared with the vehicle-treated group (*p*<0.01).

### Effects of KRS and KGS on Brain Infarction

**Figure 3:**
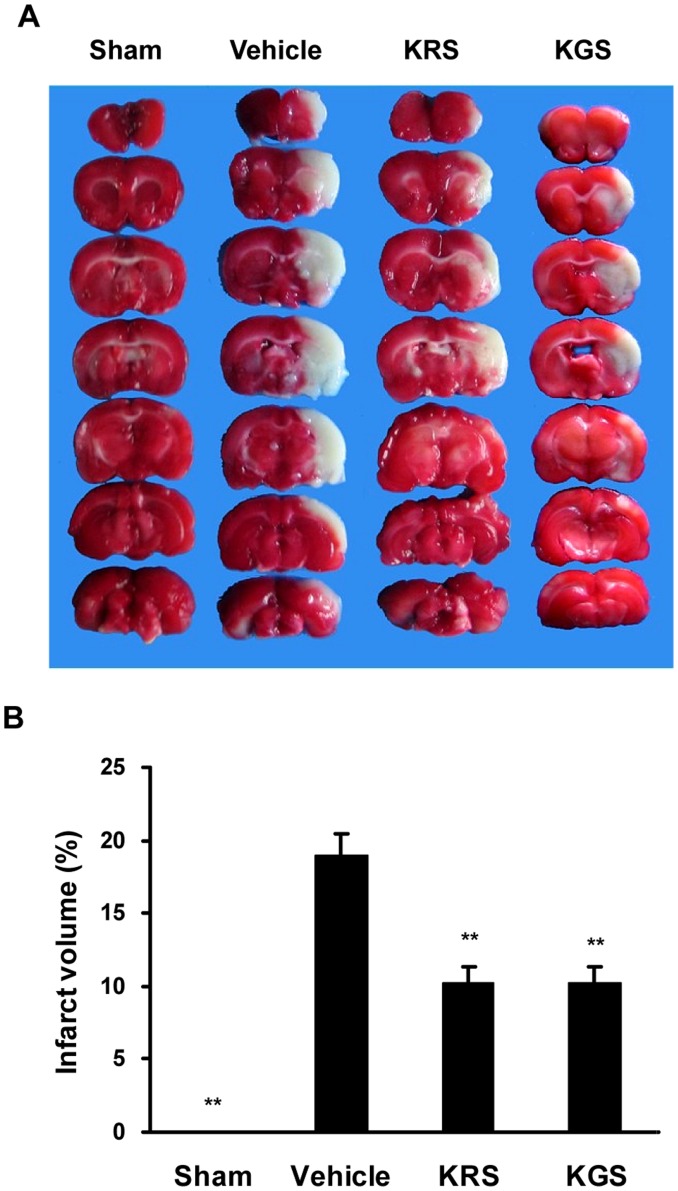
KRS and KGS attenuated brain infarction in tMCAO rats. (A) Representative photographs of TTC-stained brain coronal sections. (B) Infarct volume expressed as a percentage of whole brain volume. Treatment with KRS or KGS greatly ameliorated the infarct volume compared with the vehicle-treated controls. The data are expressed as mean ± SEM (*n  = *8 each group). *p<0.05, **p<0.01, compared with the vehicle-treated group.

Figure 3A shows the brain infarct area in each group after 22 h reperfusion following 2 h MCAO. Because the rats in the sham-operated group underwent a similar surgical procedure but were not subjected to MCAO, no infarct area was found in this group. In the vehicle-treated rats, however, the ischemic zone was identified as a distinct pale-stained area in the right cortex and striatum in the ipsilateral ischemic hemisphere (Figure 3A), with an infarct volume of 18.8±1.4% of the entire brain volume. In agreement with the neurobehavioral evaluation, post-ischemic treatment with KRS (10.0 mg/kg) or KGS (7.5 mg/kg) markedly reduced the infarct volume to 10.2±1.1% and 10.1±1.1%, respectively, compared with the vehicle-treated group (*p<*0.01; Figure 3B).

### Neuron and Axon Damage

**Figure 4:**
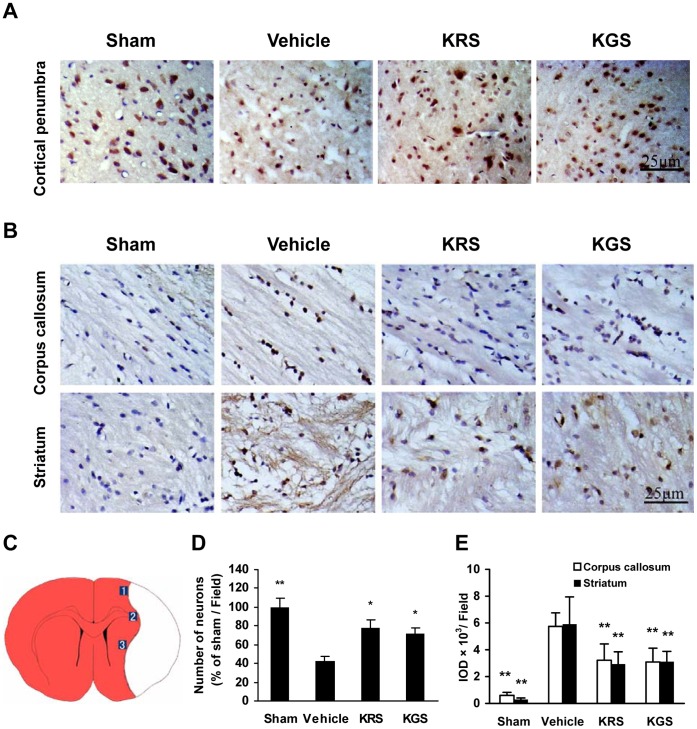
KRS and KGS attenuated ischemic neuron and axon damage in tMCAO rats. (A) Representative photomicrographs of NeuN-immunopositive neurons (brown staining) in the cortical ischemic penumbra. The neurons injured by ischemia had altered morphology or died. (B) Representative photomicrographs of APP immunoreactivity (brown staining) in the ipsilateral corpus callosum and striatum. The damaged axons were intensely stained for APP. (C) Schematic representation of a coronal brain section shows the ischemic penumbra in cortex, corpus callosum and striatum. Three fields were selected at each indicated position for quantitative measurements of immunohistochemical staining. (D) Relative number of NeuN-positive neurons expressed as a percentage of the neurons in the sham-operated group. (E) APP immunoreactivity measured using the integrated optical density (IOD) of the immunostained-positive cells. Treatment with KRS or KGS significantly reduced neuron and axon damage compared with the vehicle-treated controls. The data are expressed as mean ± SEM (*n  = *6 each group). *p<0.05, **p<0.01, compared with the vehicle-treated group.

Damage to neurons and axons after ischemia-reperfusion was examined by NeuN and APP immunostaining, respectively. Figure 4A and B show that the sham-operated rats had a large number of NeuN-immunopositive neurons in the cerebral cortex and very low APP immunoreactivity in the corpus callosum and striatum. In the vehicle-treated group, however, the neuronal cells in the cortical penumbra clearly exhibited the characteristic morphological features of ischemic damage, including shrinkage, triangulation and loss (by 57.2% compared with the sham group, *p*<0.01; Figure 4A, C), whereas the axonal damage was identified by greatly increased APP immunoreactivity in the ipsilateral corpus callosum and striatum (*p*<0.01 compared with the sham group; Figure 4B, D). Treatment with KRS or KGS increased NeuN-positive neurons to 77.8±8.9% and 71.9±5.6% of the sham group in the cortical ischemic penumbra, respectively, and greatly decreased APP expression in the ischemic corpus callosum and striatum compared with the vehicle-treated group (*p*<0.05 or *p*<0.01).

### Neuropathological Changes in Microglia and Astrocytes

**Figure 5:**
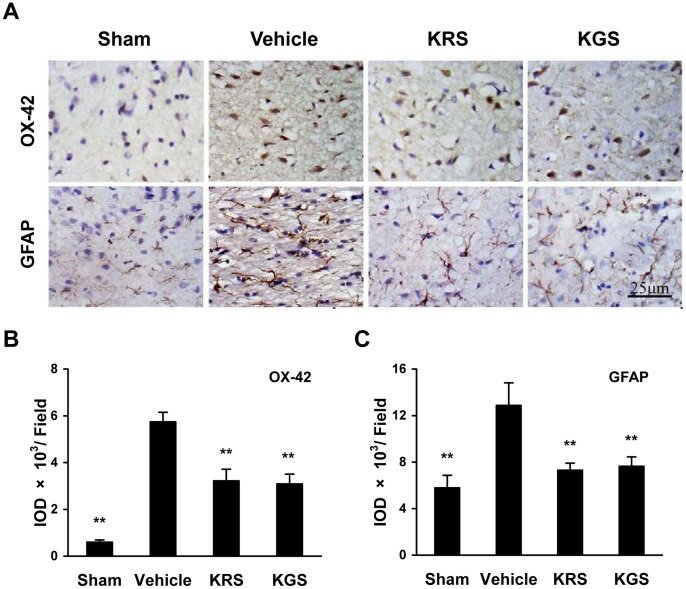
KRS and KGS improved the neuropathological changes of microglia and astrocytes in tMCAO rats. (A) Representative photomicrographs of OX-42-positive microglia and GFAP-positive astrocytes (brown staining) in the cortical ischemic penumbra. The activation of microglia and astrocytes induced by ischemia were recognized as increased expression of OX-42 and GFAP, respectively, as well as altered morphology. (B, C) The immunoreactivity of OX-42 or GFAP measured using the integrated optical density (IOD) of the immunostained-positive cells. Treatment with KRS or KGS significantly inhibited the activation of microglia and astrocytes compared with the vehicle-treated controls. The data are expressed as mean ± SEM (*n  = *6 each group). **p<0.01, compared with the vehicle-treated group.

Immunostaining for OX-42 and GFAP was used to assess the pathological changes of microglia and astrocytes, respectively, which are involved in the neuroinflammatory processes after cerebral ischemia-reperfusion. Figure 5 shows that there were only a few OX-42- and GFAP-immunoreactive cells as well as very low immunoreactivity of OX-42- and GFAP in the cerebral cortex in the sham-operated group. The vehicle-treated rats had strikingly increased number of OX-42- and GFAP-positive cells observed in the cortical ischemic penumbra, accompanied by the activated morphology of OX-42-positive microglia, which were significantly decreased by KRS or KGS treatment compared with the vehicle-treated group (*p*<0.01; Figure 5B, C).

### Activation of NF-κB and STAT3

**Figure 6:**
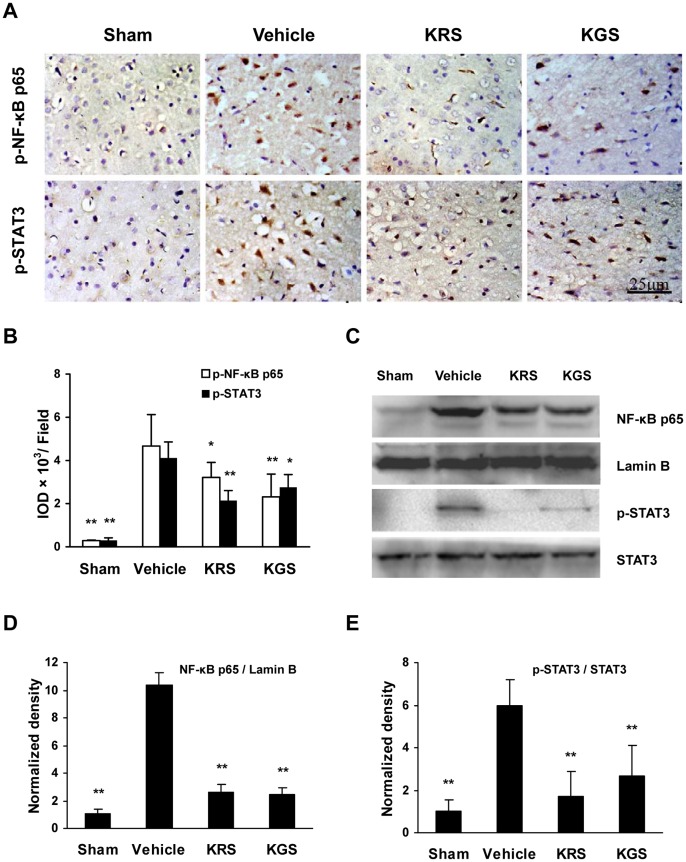
KRS and KGS inhibited the activation of NF-κB and STAT3 in tMCAO rats. (A) Representative photomicrographs of the immunohistochemical expression of p-NF-κB p65 and p-STAT3 (brown staining) in the cortical ischemic penumbra. (B) Immunoreactivity of p-NF-κB p65 and p-STAT3 measured using the integrated optical density (IOD) of the immunostained-positive cells (*n  = *6 each group). (C) Representative immunoblots of NF-κB p65, Lamin B, p-STAT3, and STAT3. The data are expressed as mean ± SEM. (D, E) Immunoblot expression measured using densitometry. The data are expressed as mean ± SEM (*n  = *3 each group). The loaded protein amount was normalized to Lamin B or STAT3, and the data are expressed as a percentage of the mean value in the sham-operated group. Treatment with KRS or KGS significantly reduced the phosphorylation of STAT3 at Tyr705, phosphorylation of NF-κB p65 at Ser536 and the nuclear content of NF-κB p65. *p<0.05, **p<0.01, compared with the vehicle-treated group.

The activation of transcription factors NF-κB and STAT3 was assayed by examining the phosphorylated levels of NF-κB p65 at Ser536 (p-NF-κB p65) and STAT3 at Tyr705 (p-STAT3) using immunostaining, and the nuclear content of NF-κB p65 and p-STAT3 level using Western blot. The immunoreactivity of p-NF-κB p65 and p-STAT3 was not significantly detected in the sham-operated group, which was strongly increased *in situ* in the ipsilateral cortical ischemic penumbra in vehicle-treated group (Figure 6A, B). Moreover, the Western blot results showed increased levels of p-STAT3 in tissue extracts and NF-κB p65 in nuclear extracts from the ischemic cortex in the vehicle-treated group compared with the sham-operated group (Figure 6C). However, treatment with KRS and KGS inhibited the phosphorylation of STAT3 and NF-κB p65 as well as the nuclear translocation of NF-κB p65 compared with the vehicle-treated group (*p*<0.05 or *p*<0.01; Figure 6D, E **)**.

### Expression of Proinflammatory Mediators

**Figure 7:**
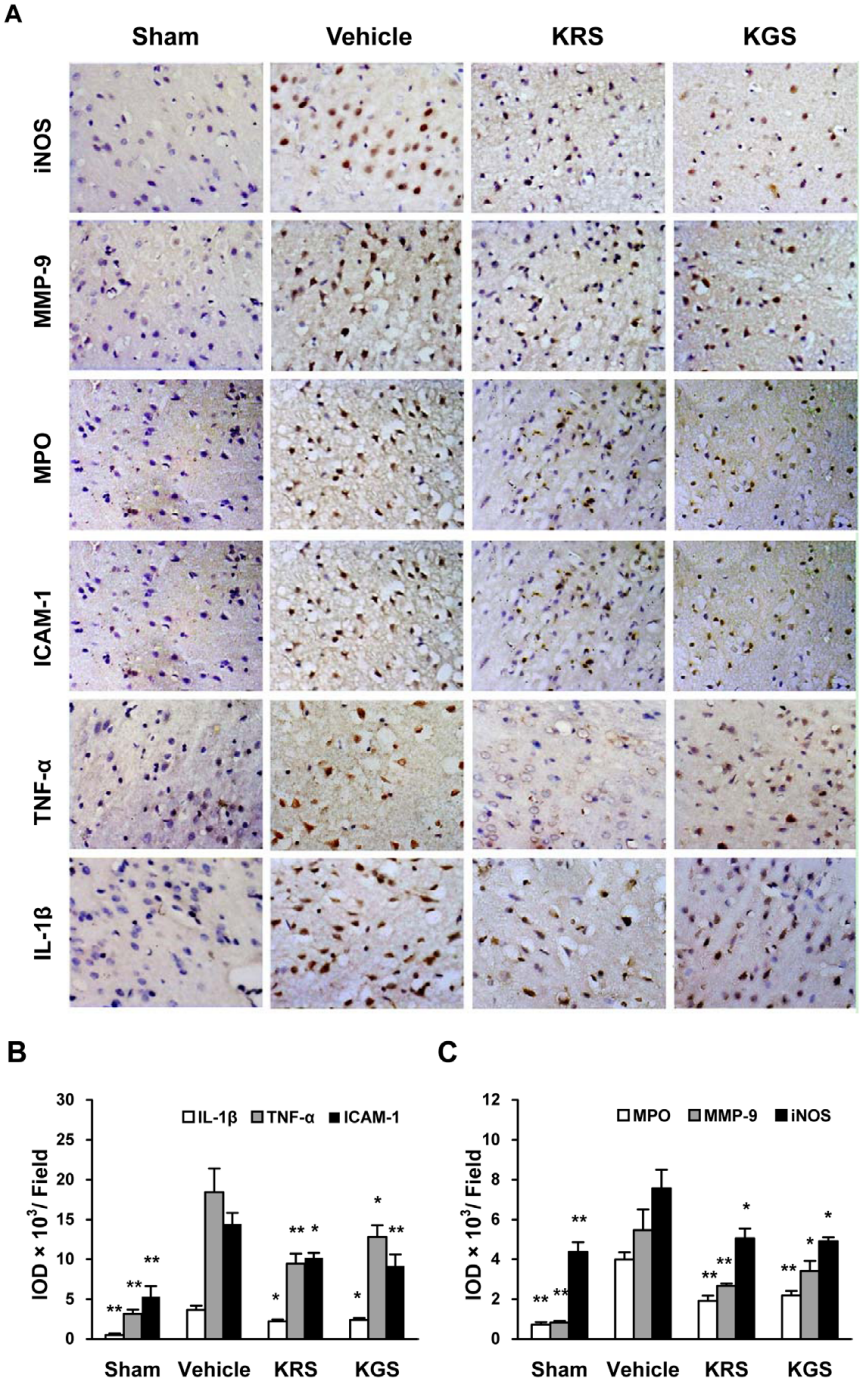
KRS and KGS inhibited the expression of proinflammatory mediators in tMCAO rats. (A) Representative photomicrographs of the immunohistochemical expression of iNOS, MMP9, MPO, ICAM, TNF-α, and IL-1β (brown staining) in the cortical ischemic penumbra. (B, C) Immunoreactivity of proinflammatory mediators measured using the integrated optical density (IOD) of the immunostained-positive cells. The data are expressed as mean ± SEM (*n  = *6 each group). Treatment with KRS and KGS significantly reduced the expression of proinflammatory mediators. *p<0.05, **p<0.01, compared with the vehicle-treated group.

To assess the effects of KRS and KGS on the neuroinflammatory response induced by tMCAO, immunostaining was used to detect the expression of the main proinflammatory mediators regulated by NF-κB and STAT3 activation as well as the neutrophil infiltration. The immunoreactivity of inflammatory enzymes (iNOS, MMP-9, and MPO), adhesive molecule ICAM-1, and cytokines (TNF-α and IL-1β) was not significantly detected in the sham-operated group, whereas the immunoreactivity of these proinflammatory mediators strongly increased in the cortical ischemic penumbra in the vehicle-treated group compared with the sham-operated group (Figure 7A **)**. However, treatment with KRS or KGS significantly attenuated the increased expression of these proinflammatory factors in the cortical ischemic penumbra compared with the vehicle-treated group (*p<*0.05 or *p<*0.01, Figure 7B, C), which indicated that KRS or KGS inhibited the ischemic neuroinflammatory process regulated by NF-κB and STAT3 activation as well as the neutrophil accumulation.

## Discussion

Intravenous administration is generally the preferred clinical approach for the treatment of acute ischemia stroke. Kaempferol-3-*O*-glucoside (KGS) and kaempferol-3-*O*-glucoside (KGS) have relatively higher water solubility than kaempferol without a sugar moiety. The previous study showed that kaempferol inhibited lipopolysaccharide-induced inflammatory response in cultured BV2 microglial cells [22]. Herein, we studied the effects of intravenous injection of KRS or KGS on neuroinflammation and brain injury in tMCAO rats.

Middle cerebral artery infarction is found in nearly 60% of clinical patients with cerebral infarction. Therefore, cerebral injury induced by tMCAO is the most relevant model of human stroke [2]. Intravenous KRS administration after the onset of ischemia reduced cerebral infarct volume and neurological deficit scores in tMCAO rats [12]. In the present study, we further evaluated the neuroprotection induced by KRS administered after the onset of reperfusion following ischemia in tMCAO rats. We then compared the efficacy of KRS to another flavonoid glycoside, KGS. At the end of 2 h MCAO, the rats with neurological scores ≥2 were randomly divided into the different treatment groups to ensure the comparability of cerebral postischemic outcome. We found that treatment with equimolar doses of KRS (10 mg/kg) and KGS (7.5 mg/kg) similarly and significantly ameliorated cerebral infarction and neurological deficits compared with the vehicle-treated group. To our knowledge, this is the first report of the neuroprotective effect of KGS against ischemic brain damage.

To further determine the neuroprotective role of KRS and KGS in acute ischemic stroke, we examined the effects of these flavonoid glycosides on ischemia-reperfusion injury in both gray matter neurons and white matter axons using immunohistochemistry. NeuN, a specific marker of mature neurons, has been widely used to determine neuronal death or survival in ischemic brain tissue [23]. APP, a marker of disrupted axonal flow marker, is regarded as a key predictor of axonal injury in central nervous system diseases [24]. Consistent with previous studies, we found that cerebral ischemia-reperfusion significantly induced neuron loss in the cerebral cortex and axonal damage in the corpus callosum and striatum in the ipsilateral ischemic hemisphere [23], [25]. With KRS and KGS treatment, however, the number of NeuN-positive neurons was markedly increased, and APP expression was significantly decreased. Our results showed that postischemic treatment with these kaempferol glycosides prevented the neuron and axon damage after cerebral ischemia-reperfusion.

Numerous studies have shown that cerebral ischemia-reperfusion not only damages neurons and axons but also affects all types of glial cells, including microglia, astrocytes, and oligodendrocytes. The glial response to cerebral ischemia-reperfusion is involved in the evolution of ischemic brain damage [26∼28]. Therefore, we examined the effects of KRS and KGS on specific markers of glial cells following ischemia-reperfusion using immunohistochemistry (i.e., OX-42 for activated microglia; GFAP, an astrocyte-specific cytoskeletal protein, for reactive astrocytes). We found that postischemic administration of KRS and KGS significantly reduced the expression of OX-42 and GFAP in the ipsilateral cortex. Our findings suggest that the neuroprotective effects of these kaempferol glycosides are associated with inhibitory effects on the glial response to cerebral ischemia-reperfusion.

In the brain, glial cells bimodally contribute to the pathophysiological events of neuroinflammation. Microglial activation, for example, is normally necessary for scavenging necrotic debris and other exogenous substances, but the overactivation of microglia and reactive astrocytes after ischemia-reperfusion may promote the activation of proinflammatory transcription factors and subsequent production of many proinflammatory mediators [3, 5, 28). Of these, the activation of STAT3 and NF-κB plays a predominant role in the neuroinflammatory cascades and secondary cerebral injury after ischemic stroke by controlling the expression of many proinflammatory genes [5]. STAT proteins were identified as critical transcription factors that mediate virtually all cytokine-driven signaling [29]. Of the various STAT isoforms, STAT3 is the most-conserved isoform, and phosphorylation at Tyr705 is required for STAT3 activity. After phosphorylation at Tyr705, dimerization, and nuclear translocation, STAT3 binds to DNA to induce the expression of many genes that contribute to neuroinflammation and brain damage after focal ischemia [30], [31]. Additionally, NF-κB comprises a family of transcription factors that consists of five different proteins–p50, RelA/p65, c-Rel, RelB, and p52–that can combine to form active dimers in response to ischemic insult. NF-κB activation is mainly mediated by the IκB degradation-dependent pathway and a novel IκB degradation-independent pathway that involves the phosphorylation of p65 at Ser536 [32], [33]. By inducing the degradation of IκB proteins, the classic pathway results in the liberation of the p65/p50 heterodimer and translocation of this protein into the nucleus, where the p65/p50 complexes bind to target sites and induce the transcription of proinflammatory mediators. Consistent with previous data, our study showed that the phosphorylation of STAT3 at Tyr705 and NF-κB p65 at Ser536 was significantly increased in the ischemic cortical penumbra. Moreover, immunoblotting revealed an increase in the nuclear content of NF-κB p65 and STAT3 phosphorylation in the tissue extracts of the ischemic cortex. Therefore, cerebral ischemia-reperfusion induced the activation of STAT3 and NF-κB, including both the independent and dependent pathways of IκB degradation. Accordingly, the expression of main proinflammatory genes, including TNF-α, IL-1β, iNOS, MMP-9, and ICAM-1, was significantly increased in the ipsilateral cortical penumbra. However, treatment with KRS and KGS strongly inhibited the activation of STAT3 and NF-κB in the ipsilateral ischemic region, thereby repressing the subsequent expression of proinflammatory mediators.

Furthermore, neutrophil infiltration in the ischemic brain plays an important role in secondary inflammatory injury after stroke. A previous study found that neutrophils were the first inflammatory cells to arrive at ischemic brain tissue, as early as hours after reperfusion [34]. After the upregulation of adhesion molecule expression (e.g. ICAM-1) at the vascular endothelium and breakdown of BBB integrity, with dysfunction of the reconstruction of the extracellular matrix following increased MMP-9 expression, neutrophils may migrate from blood to brain parenchyma where they may further increase the degree of cerebral ischemia by obstructing microvessels and releasing inflammatory mediators [35], [36]. To explore whether the neuroprotective effect of kaempferol glycosides is related to the inhibition of neutrophil infiltration after cerebral ischemia-reperfusion, we examined the effects of KRS and KGS on the expression of MPO, a specific neutrophil marker. We observed a very high level of MPO immunoreactivity in cerebral ischemic penumbra in tMCAO rats, whereas treatment with KRS and KGS strongly inhibited MPO expression. Considering a previous report in which KRS induced the production of NO by upregulating eNOS activity and maintaining regional cerebral blood flow (CBF) in tMCAO rats [12], the present study further suggests that KRS and KGS likely prevented neuroinflammatory injury by improving neurovascular dysfunction after cerebral ischemia-reperfusion.

In summary, postischemic treatment with KRS and KGS prevented the neurobehavioral deficits, cerebral infract volume, neuron and axon damage, and neuropathological response of glial cells (i.e., microglia, astrocytes, and oligodendrocytes) induced by transient focal cerebral ischemia in rats. The mechanism of these kaempferol flavonoid effects appears to be at least partially associated with antineuroinflammatory effects by inhibiting the activation of STAT3 and NF-κB p65, including independent and dependent pathways of IκB degradation, and subsequent expression of proinflammatory mediators. Our findings suggest that postischemic treatment with KRS or KGS attenuates cerebral ischemia-reperfusion injury and neuroinflammation by inhibiting the activation of STAT3 and NF-κB and has the therapeutic potential for the neuroinflammation-related diseases, such as ischemic stroke.
