# Correction: Neuroprotective effect of Kaempferol Glycosides against brain injury and Neuroinflammation by inhibiting the activation of NF-κB and STAT3 in transient focal stroke

**DOI:** 10.1371/journal.pone.0320685

**Published:** 2025-03-12

**Authors:** Lu Yu, Chu Chen, Liang-Fen Wang, Xi Kuang, Ke Liu, Hao Zhang, Jun-Rong Du

The MPO and ICAM-1 rows in Fig 7A of this article [1] appear similar due to an error in the published figure.

**Fig 7:**
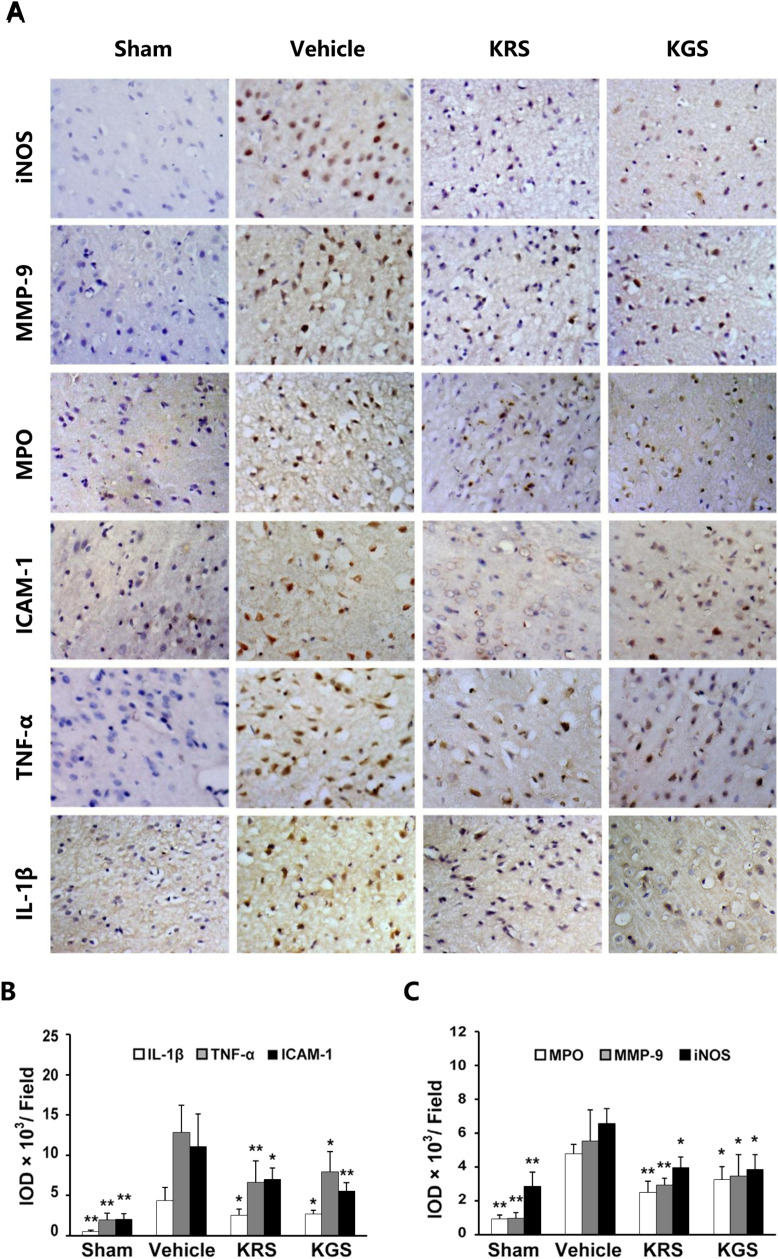
KRS and KGS inhibited the expression of proinflammatory mediators in tMCAO rats. (A) Representative photomicrographs of the immunohistochemical expression of iNOS, MMP9, MPO, ICAM, TNF-α, and IL-1β (brown staining) in the cortical ischemic penumbra. (B, C) Immunoreactivity of proinflammatory mediators measured using the integrated optical density (IOD) of the immunostained-positive cells. The data are expressed as mean ±  SEM (*n* =  6 each group). Treatment with KRS and KGS significantly reduced the expression of proinflammatory mediators. * *p* < 0.05, **p < 0.01, compared with the vehicle-treated group.

The authors provide the correct version of Fig 7 here where the results presented for multiple experimental conditions have been updated as follows:

- Data reported in the published article for TNF-α are now reported as representing results for ICAM-1.
- Data reported in the published article for IL-1β are now reported as representing results for TNF-alpha.
- New images from the original experiments have been provided in the IL-1β panels.

The authors provided underlying images for all panels in Fig 7A, including replication data from the time of the original experiments (S1–S6 Files).

The immunoreactivity IOD of IL-1β, TNF-α, and ICAM-1 were recalculated based on the original images (S1–S6 Files) using ImagePro Plus 6.0 software. The authors noted that there were slight changes among the three IOD data sets compared to the previous values calculated by ImagePro Plus 5.0 software in Figs 7B and 7C in [1]. Therefore, the authors also recalculated the immunoreactivity IOD of the other three markers (MPO, MMP-9, and iNOS). The individual-level underlying data for the updated Figs 7B and 7C are in S7 File.

A member of *PLOS One*’s Editorial Board reviewed the updated Fig 7 and advised that it supports the results and conclusions as reported in the original article [1].

The remainder of the data underlying this article [1] are available from the corresponding author.

## Supporting information

S1 File Underlying ICAM images supporting Fig 7A, including replication data from the time of the original experiments. (RAR)

S2 File Underlying IL-1β images supporting Fig 7A, including replication data from the time of the original experiments. (RAR)

S3 File Underlying iNOS images supporting Fig 7A, including replication data from the time of the original experiments. (RAR)

S4 File Underlying MMP9 images supporting Fig 7A, including replication data from the time of the original experiments. (RAR)

S5 File Underlying MPO images supporting Fig 7A, including replication data from the time of the original experiments. (RAR)

S6 File Underlying TNFα images supporting Fig 7A, including replication data from the time of the original experiments. (RAR)

S7 File Underlying quantitative data supporting Figs 7B and 7C. (XLSX)
